# Three-Dimensional Immersive Photorealistic Layered Dissection of Superficial and Deep Back Muscles: Anatomical Study

**DOI:** 10.7759/cureus.26727

**Published:** 2022-07-11

**Authors:** Toma Spiriev, Atanas Mitev, Viktor Stoykov, Nikolay Dimitrov, Ivan Maslarski, Vladimir Nakov

**Affiliations:** 1 Department of Neurosurgery, Acibadem Cityclinic University Hospital Tokuda, Sofia, BGR; 2 Anatomy, Department of Anatomy and Histology, Pathology and Forensic Medicine, University Hospital Lozenetz, Medical Faculty, Sofia University, Sofia, BGR; 3 Department of Neurosurgery, Acibadem City Clinic Tokuda Hospital, Sofia, BGR

**Keywords:** plastic surgery, virtual reality, augmented reality, back muscles, neurosurgery, anatomy, surface scanning, photogrammetry

## Abstract

Introduction

The distinct anatomy of the superficial and deep back muscles is characterized by complex layered courses, fascial planes, specific vascularization, and innervation. Knowledge of these anatomical parameters is important for some surgical approaches, including lumbar disc herniation, cerebrospinal fluid fistula repair, vascularized muscle pedicle flaps, and posterior fossa extra-intracranial bypass. In the present study, we use modern techniques of three-dimensional (3D) surface scanning to help better illustrate the layered anatomy of the back muscles.

Material and methods

We dissected in layers* the back muscles of one cadaver*. Every step of the dissection was 3D scanned using a technique called photogrammetry, which allows the extraction of 3D data from 2D photographs. The 3D data were processed using Blender software, and the 3D photorealistic models were uploaded to a dedicated website for 3D visualization. This allows users to see the 3D models from every desktop or mobile device, as well as augmented (AR) and virtual reality (VR) formats.

Results

The photorealistic 3D models present the back muscles' anatomy in a volumetric manner, which can be visualized on any computer device. The web 3D features, including AR and VR, allow users to zoom, pan, and rotate the models, which may facilitate learning.

Conclusion

The technology of photorealistic surface scanning, modern 3D visualization possibilities of web-dedicated formats, as well as advances in AR and VR, have the potential to help with a better understanding of complex anatomy. We believe that this opens the field for further research in the field of medical education.

## Introduction

The gross human anatomy of the back muscles is distinct with specific layered structure, particular innervation, and vascularization depending on the type of muscle: extrinsic (superficial) or intrinsic (deep) back muscles. This layered structure can be difficult to understand when studied from traditional two-dimensional (2D) atlases because one cannot easily recognize the complex three-dimensional (3D) relationships of the soft tissue layers, individual muscle courses, intermuscular fascial planes, muscular attachments, and the related neurovascular anatomy from flat photographs and schemes presented from only one angle of view.

Recently, there has been a growing trend of publications of 3D anatomical models that are created with the help of different methods of surface scanning techniques [1,2]. One such technique, called photogrammetry, is one of the ways to create such photorealistic 3D models. Photogrammetry consists of a process of surface scanning that utilizes standard photographic equipment for the generation of volumetric 3D data [1-3]. This process is highly resource-intensive in terms of computational power, but if the photographs are captured using a modern high-megapixel camera and post-processed accordingly, the result can represent a highly accurate photorealistic 3D model. Photogrammetry is widely used in geography in order to generate a 3D landscape from low-altitude aerial photographs [4,5] or in archeology in order to 3D map excavations or create 3D spatial reconstructions of historical monuments [6]. Neurosurgery is a relatively new application field for this technology with relatively few studies, mainly for skull base surgery and brain anatomy [3-7].

In the current paper, we use photogrammetry to create photorealistic 3D models of the back muscles' anatomy and explore the possibilities that modern technology allows to present these data through desktop and mobile devices or in the form of augmented reality (AR) and virtual reality (VR). Despite the lack of solid evidence that learning from computer-based 3D atlases and VR atlases is more efficient than traditional teaching, we believe that such 3D models can be used for medical education in the fields of anatomy, neurosurgery, and plastic surgery and potentially facilitate the study process due to the interaction and "first-person experience" that 3D models provide [7,8]. The aim of the current study is to demonstrate the benefits of photogrammetry technology in the field of anatomy, which can potentially facilitate the creation of 3D models by non-professional 3D artists and will make 3D technology more available to the general medical community.

## Materials and methods

We dissected in layers the back muscles of one cadaver, respecting ethical and legal standards. Initially, for the first models, we used the following process: every step of the dissection was photographed from multiple angles using an eight-megapixel Canon 350D DSLR camera. For the purpose of representative photorealistic models, between 70 and 120 photographs were taken. The photographs were taken in RAW format, which contains uncompressed image data and allows much more detailed images of the anatomical specimen. The *.RAW files of the photographs were then uploaded to Darktable software (https://www.darktable.org), which is an open-source photography workflow application and RAW developer, allowing for multiple image processing. The images were extracted in 8/16-bit TIFF or JPEG and transferred to Agisoft Metashape software v.1.7.5 (Agisoft LLC, St. Petersburg, Russia), which is a dedicated photogrammetry software. The workflow in this software was the following: first the images were imported to the software and aligned, which is a process allowing the program to estimate the position where the photographs were taken in space. Next, a dense cloud was built where common surface points were extracted from photos and clouds of colorized coordinates were created. After this step, the points were triangulated and a 3D mesh was built. The final step was the texture generation, and the model was ready to be exported from the program in the form of a *.obj file.

As an alternative to this long process for the later 3D models, we used mobile phone-based software called Metascan (Abound Labs Inc., New York, NY, USA), where the photos were taken in a similar way described above, but the whole photogrammetry computing process is cloud-based and is not done on the mobile phone. After the cloud processing was finished and the data were sent back to the device, the ready 3D model was finally exported as a *.fbx file.

**Figure 1:**
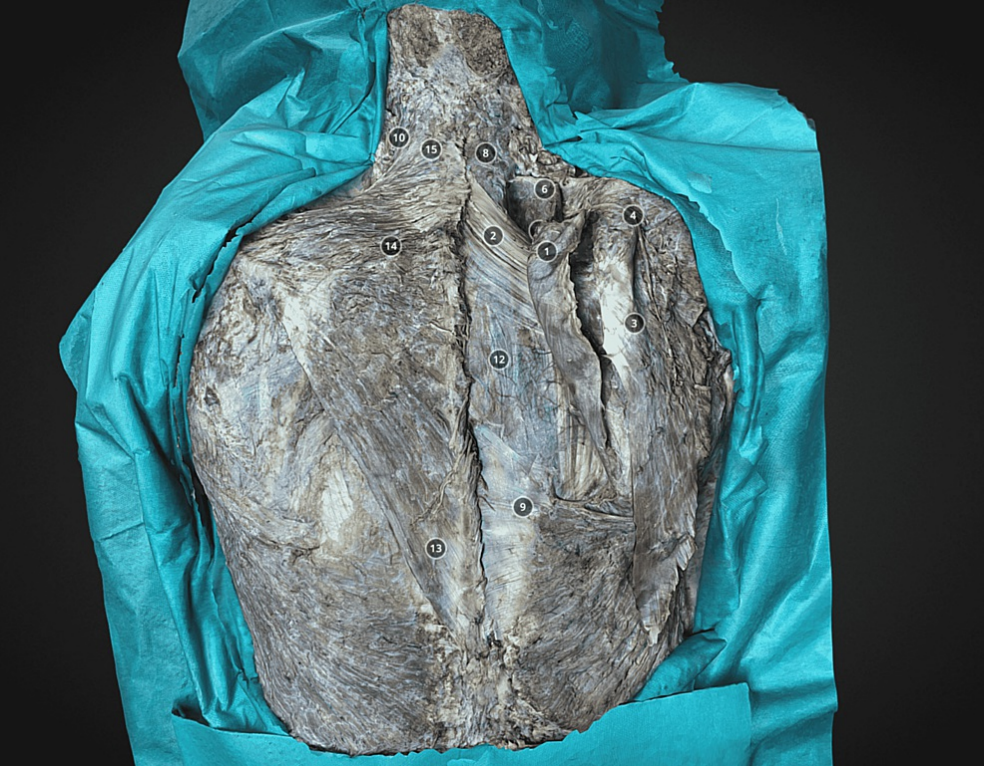
Example of superficial (extrinsic) back muscles dissection. Rhomboid muscles are mobilized and reflected (1). The serratus posterior superior muscle is revealed (11) on the right side. The left side the more superficial muscles such as trapezius (5), latissimus dorsi m. (14) teres minor (16) and teres major m. (17) are presented on the left side. Levator scapulae muscle (9) is reflected and its innervation is revealed (dorsal scapular nerve (10)). Model 3: https://sketchfab.com/3d-models/3-layered-anatomy-back-muscles-dissection-339a5b701ea94eafa2fa3ed12d955685.

The 3D photorealistic model was further processed using Blender software 2.92 (Blender Foundation, Amsterdam, Holland), which is an open-source 3D computer graphics software toolset. With the help of this software, the unnecessary parts of the models (mainly the environment, which is not important to the model) were removed. The final 3D model was exported to the Sketchfab platform (Sketchfab; Sketchfab Inc, New York, NY, USA), which is an Internet 3D viewer based on the Web Graphics Library (WebGL) technologies that allow users to display 3D models on the web, and later view them on any mobile browser or in the form of AR or VR with a dedicated headset. On the Sketchfab platform, the 3D models are annotated. Every relevant structure (muscle, vessels, or nerves) was pointed out (Figure 1).

The VR feature of the Sketchfab platform was tested using the Oculus Quest 2 VR headset (Facebook Technologies, LLC., https://www.oculus.com/).

## Results

**Figure 2:**
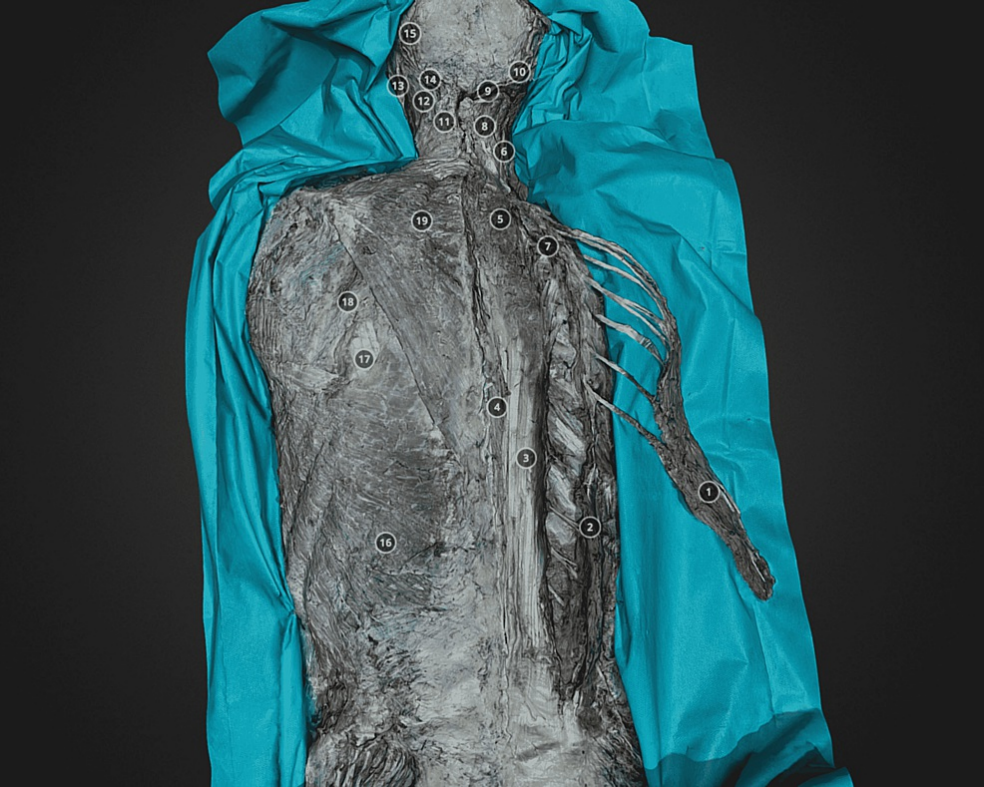
Deep (intrinsic) back muscles dissection. The iliocostalis m. is mobilized and its attachments are presented. The individual muscles of the erector spinae muscle group are presented. https://sketchfab.com/3d-models/9-illiocostalis-m-mobilised-432130b7916a465d93597456b3c09f6a.

Eleven 3D models were generated that showed the back's extrinsic (superficial) and intrinsic (deep) muscles (Figures 1-2).

The models were numbered sequentially, and the weblinks corresponding to each model are presented in Table 1.

**Table 1:** Presentation of all 3D models with their descriptions and associated weblinks.

3D model number	Description	Weblink to the 3D model
Model 1	Superficial muscle anatomy of the back—extrinsic (superficial) muscles	https://sketchfab.com/3d-models/1-superficial-muscle-anatomy-of-the-back-ceb327afc9a142d8809c197d6e1dd0e0
Model 2	Right trapezius muscle is mobilized and reflected laterally showing its innervation (accessory nerve)	https://sketchfab.com/3d-models/2-anatomy-dissection-of-the-back-muscles-a445270875334764b46a125351371013
Model 3	Rhomboid muscles are mobilized and reflected	https://sketchfab.com/3d-models/3-layered-anatomy-back-muscles-dissection-339a5b701ea94eafa2fa3ed12d955685
Model 4	Trapezius, splenius capitis, and cervicis muscles are reflected laterally	https://sketchfab.com/3d-models/4-layered-back-muscles-dissection-016e7691af564ac494eb57b6a5c93c12
Model 5	The semispinalis capitis muscle is reflected and the semispinalis cervicis muscle as well as the occipital triangle are presented	https://sketchfab.com/3d-models/5-anatomy-of-back-muscles-2641390f57c44da0b5e348d30b0baf08
Model 6	The latissimus dorsi muscle is fully dissected and presented	https://sketchfab.com/3d-models/6-latissimus-dorsi-muscle-presented-7eb4c44c64df490bb50e88b05e9a88a5
Model 7	The right latissimus dorsi muscle is mobilized and reflected revealing its innervation and vasculature (thoracodorsal artery and nerve)	https://sketchfab.com/3d-models/7-latissimus-reflected-02a390ee94bc4a57b5ac88c3558c41ce
Model 8	Erector spinae muscles presented	https://sketchfab.com/3d-models/8-erector-spinae-muscles-c4df9386b7ef4f4ba4d0953879f33518
Model 9	The iliocostalis m. is mobilized and its attachments are presented	https://sketchfab.com/3d-models/9-illiocostalis-m-mobilised-432130b7916a465d93597456b3c09f6a
Model 10	Longissimus muscle reflected	https://sketchfab.com/3d-models/10-longissimus-muscle-reflected-ac7acfd7101542fa9ae9a54d5d5bee8f
Model 11	The deepest muscles of the back are presented - suboccipital triangle and multifidus mm.	https://sketchfab.com/3d-models/11-semispinalis-m-reflected-38230823060743e5ab6b76748b3854be

The right side was primarily dissected in order to compare left and right anatomical regions and perceive the depth of the dissection. Each anatomical structure of interest was numbered and annotated. Upon choosing the relevant structure, the camera is automatically directed to it. There is a drop-down list of all the annotations in the middle of each 3D model. First, this skin and subcutaneous tissue were removed, revealing the first layer of the superficial muscles-the trapezius, latissimus dorsi, part of the splenius, and the rhomboid muscles (Model 1). Later, the trapezius muscle was mobilized and reflected, presenting its innervation by the accessory nerve and vascularization (by the transverse cervical artery). The deeper extrinsic muscles, namely the splenius, levator scapulae, and rhomboid muscles, were exposed (Model 2). Next, the rhomboid muscles were mobilized and reflected. The serratus posterior superior muscle was revealed. The Levator scapulae muscle was reflected and its innervation was revealed (dorsal scapular nerve) (Model 3). Next, the trapezius, splenius capitis, and cervicis muscles were reflected laterally, thus revealing the semispinalis capitis muscle, as well as the longissimus muscle (Model 4). After that, the semispinalis capitis muscle was reflected, and the semispinalis cervicis muscle, as well as the occipital triangle, were presented. The vertebral artery was revealed as well as the occipitalis major nerve was visualized (Model 5). The following dissection presents the right latissimus dorsi muscle, which was mobilized and reflected, revealing its innervation and vasculature (thoracodorsal artery and nerve) (Models 6 and 7). The next models (Models 8, 9, 10, and 11) present dissection of the intrinsic (deep) muscles of the back, where sequentially the longissimus, iliocostalis, multifidus, spinalis, and semispinalis capitis muscles were presented.

**Figure 3:**
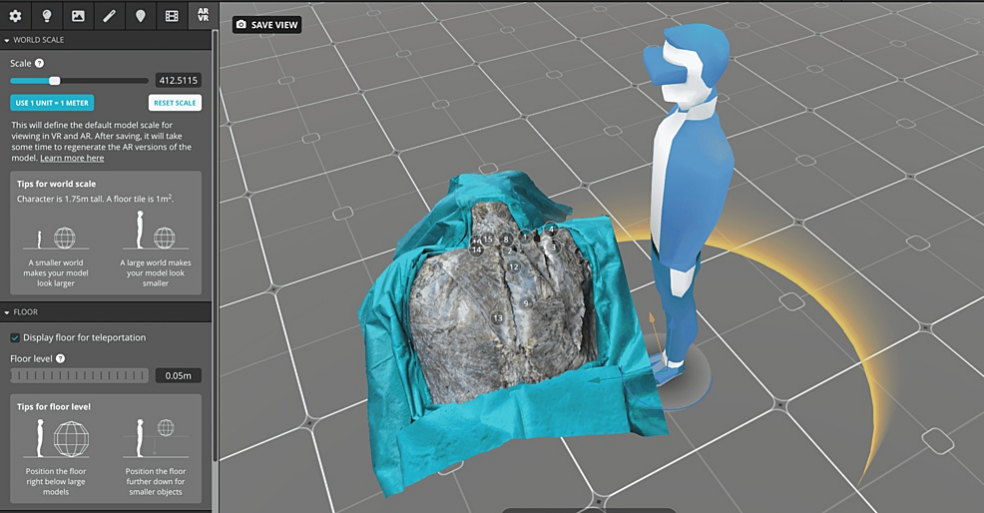
Virtual reality option within the Sketchfab editor. The observer’s position as well as the size and location of the 3D model can be modified. The observer can be “teleported” to any of the squares. The size of the 3D model can be changed in the settings and later with the VR controller.

The photorealistic 3D models allow the observer to see this complex layered anatomy of the back muscles from every possible direction on any device; zoom, pan, and rotate the models; and select annotation for the specific structure of interest. The VR settings were adjusted in the Sketchfab web editor for optimal visualization of the 3D model through the VR headset (Figure 3).

**Figure 4:**
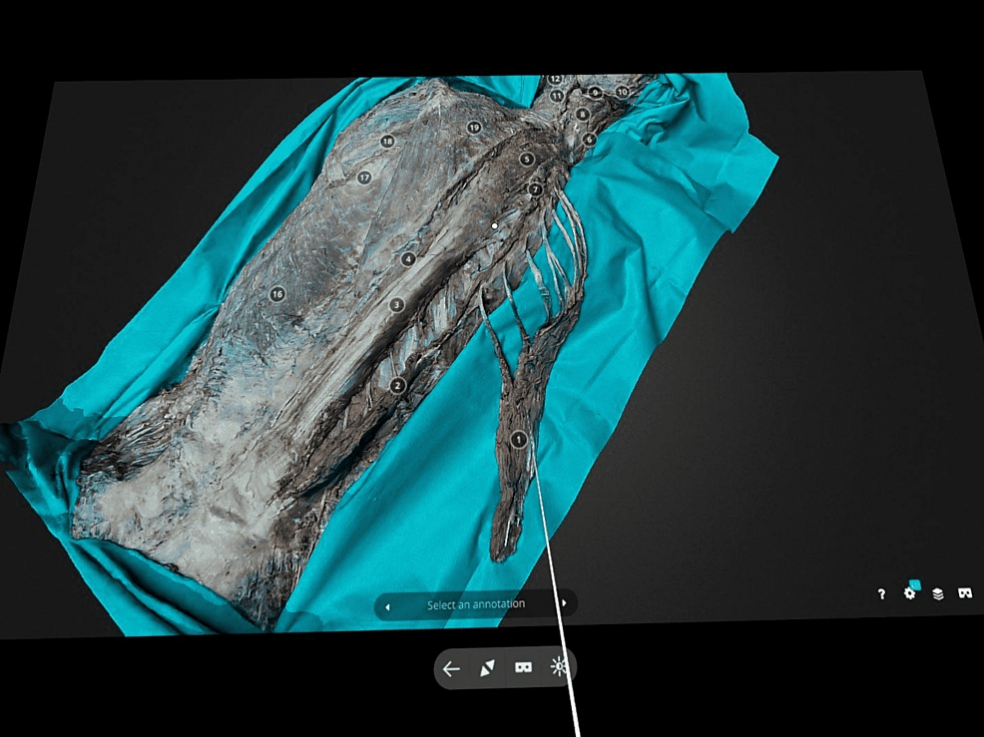
Visualization of the 3D model through the virtual reality Oculus Quest 2 stand-alone headset. The models can be viewed on a cinema like VR screen through the VR web browser.

The VR test of the technology included visualization of the models through the two different VR browsers-Oculus Browser and Firefox VR Browser-that can be downloaded from the Oculus Appstore (Figure 4).

**Figure 5:**
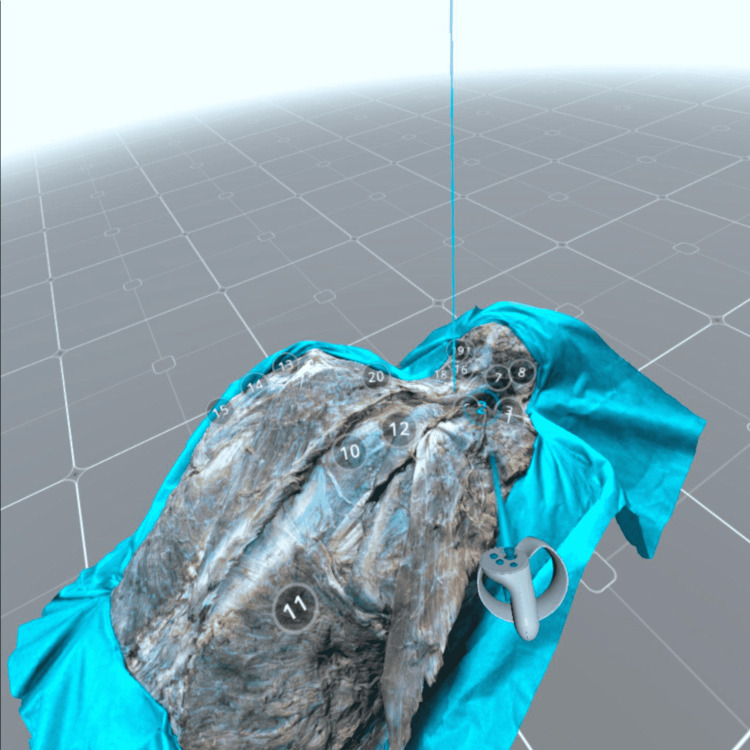
The immersive virtual reality on the Sketchfab virtual reality mode

The apps allowed two modes of visualization: a large cinema-like virtual screen or an immersive VR experience. The former allowed the models to be viewed on a very large cinema-like virtual screen, rotated from every possible angle and the annotations to be selected. Only the Firefox VR browser allowed zooming of the model. The immersive VR experience was better presented on the Oculus browser as the 3D model was better visualized and the image was sharper compared to the image on the Firefox browser. The size of the models was changed using the controller’s joystick (Figure 5).

The only limitation was that the viewing angle is fixed and can only be changed through the controller, as the observer is "teleported" to a specific viewpoint. The annotations of the anatomical structures can be selected using the hand controller but were not always visible, which is overall a small drawback compared to all the other benefits of the technology.

The AR feature was tested using the Sketchfab app on an iPhone 11 (Apple Inc., Cupertino, CA). The application allows the model to be placed anywhere on a flat surface, and the size of the model as well as the rotation can be changed with intuitive gesture control on the screen. Using the mobile phone screen, the model can be viewed from every possible angle, and zooming can be done by changing the distance to the model. There were no annotations on the relevant anatomical structures, but the combined experience from the desktop web-based viewer and VR and AR features makes learning anatomical structures a completely new experience.

## Discussion

The aim to present complex anatomy in a more comprehensive visual way has its roots at the beginning of the 20th century when the first stereoscopic atlas by Daniel John Cunningham-Stereoscopic Studies of Anatomy was published in 1905 [9,10]. This atlas required a specially designed stereoscopic viewer to examine dedicated stereoscopic anatomy photographs. Later in the century, there were other stereoscopic anatomy atlases focusing on neuroanatomy and gross human anatomy, but due to the difficulty in the production of stereoscopic photographs, there were not that many published titles [10]. In neurosurgery, the first such atlases were published in the middle of the 1980s with one distinct work by Poletti and Ojemann, which included intraoperative stereo color slides used for teaching neurosurgery residents [10,11]. This detailed work presented microscopic intraoperative anatomy as stereoscopic images. At the beginning of the 21st century, this field was pushed forward by the work of Professor Rhoton, who implemented stereoscopic anatomy in his lectures and papers [10-14]. By using first anaglyph glasses, a 3D volumetric effect was produced, where viewers could observe a 3D image through anaglyph color filtering [10-14]. Later, at the beginning of the last decade, this technology advanced with the introduction of 3D displays and stereoscopic multimedia projectors observed with active or passive 3D glasses, allowing even higher quality stereoscopic anatomy photographs and stereoscopic videos to be produced and presented to a larger audience, which set a new standard in the field of neurosurgical anatomy education [10-14]. At present, with the advances in processing power of modern computers, the development of web-based 3D platforms accessible through any mobile phone, and the slowly growing popularity of VR and AR technology, we are at the beginning of a new phase of neurosurgical training and education [10].

In our study, we have implemented a well-known methodology such as photogrammetry used primarily in geography [4,5] or archeology [6] for the generation of authentic, cadaver-based, 3D models of layered dissection of the back muscles. So far, there are relatively few studies examining the use of photogrammetry in neurosurgery, mainly in skull base surgery, brain 3D models, and cadaver dissections [15-17]. Yet, the benefits of this technology are clearly visible: the possibility to create a very accurate dissection-based 3D model that can be rotated from every possible angle, magnified, and with each important anatomical structure indicated and referenced. This process can potentially facilitate the study of complex anatomy and show accurate visuospatial structure correlation, which is one of the main disadvantages when learning from classical 2D (two-dimensional) anatomical sources-a lack of three-dimensional conceptualization and visualization of structures. The results have been consistent with many studies [18-24]. This, together with the complexity of learning neuroanatomy, called "neurophobia" by some authors [18], is one of the main reasons behind this project. Neuroanatomy, together with head and neck anatomy and pelvic anatomy, ranks highest on the difficulty scale among medical students and surgery residents [18].

Another important strength of photogrammetry-based learning is the degree of photorealism that comes with these 3D models as they are generated from actual photographs. By this way, medical professionals (students, neurosurgery residents, neuroanatomists) can study real cadaveric dissection, which, on the one hand, might be somehow more complex than conventional 3D atlases created by 3D graphic designers and 3D sculptors, where the models are very accurate and stylized, but, on the other hand, do not fully correspond to reality. In our opinion, the major weakness of the 3D graphics-based atlases is the actual tissue textures, because they are generated by complex computer algorithms and not by actual photographs.

In photogrammetry, one can get a very good result even with a relatively low-cost DSLR camera or even with the multiple camera systems of a contemporary mobile phone [25]. In our study, we used an old eight-megapixel Canon 350D DSLR camera and an iPhone 12, which resulted in acceptable and informative quality of the 3D models. This is confirmed by other authors who used high-resolution mobile phone cameras and cloud-based data processing, resulting in high-resolution 360-degree 3D models of the cadaveric specimens [15]. The authors concluded that this is an inexpensive, simple, and accessible method to create 3D models that can be used for the training of neurosurgery residents with contemporary technology such as AR and VR educational tools [15].

An important point is that photogrammetry depends on the quality of the images taken, the size of the camera sensor, depth of field, and light conditions (diffuse light is better than focused light) [3,9,25]. If the photographs are taken correctly, they are sharp, and with appropriate light conditions, the resulting textures of the 3D model can be of very high quality, even higher compared to the ones achieved with the other technology used for 3D scanning, which is structured light scanning (SLS) [3,9,25]. The latter technology uses structured light or laser-based scanners to capture surface topology, resulting in very precise 3D models that can be used for 3D printing or 3D modeling [3]. The major drawback is that structured light scanning technology requires very expensive 3D scanners; it is very demanding in terms of computational power and is typically characterized by a slow acquisition time (between 10 and 20 minutes) [3]. However, due to the precise sensors that structured light scanning uses, it allows an accuracy of approximately 100 µm, which can relate to a highly precise model, especially for deep structures such as internal acoustic meatus, foramina, canals, and other anatomical corridors [3], where photogrammetry has not produced those good results. Therefore, structured light scanning will produce the greatest geometric accuracy as it is especially useful in developing digital models for 3D printing [2].

Both techniques (SLS and photogrammetry) have their strengths and weaknesses and can be used for the creation of medical 3D models used for anatomical education. In our study, we have chosen the photogrammetry method due to its relatively low production costs, potentially better textures of the models, and the shorter amount of time required to produce a 3D model, which is an opinion shared also by other authors [3,15,25].

For our first study using the photogrammetry technique for the generation of anatomical 3D models, we chose the back muscles as this is an interdisciplinary area (neurosurgery, spine surgery, plastic surgery, physiotherapy, and kinesitherapy). The gross anatomy is relatively flat, which does not require a 360-degree scanning technique like the human skull or brain. The latter requires different photography equipment settings (rotating table, diffuse light montage, etc.). The back muscles anatomical region can be a good starting point to explore photogrammetry as a teaching tool. The muscle anatomy of the back region is distinct with a relatively complex layered course, especially at the upper thoracic and cervical levels. For neurosurgery, this anatomy is important as there are some distinct approaches and techniques where detailed knowledge of the individual muscle layers, as well as their feeding vessels, is needed-muscle splitting techniques for lumbar disc herniation [26-29], repair of cerebrospinal fluid fistulas and complex wound healing problems after posterior fossa surgery or spinal instrumentation using vascularized muscle pedicle flaps [30-34] and layered muscle dissection for posterior circulation bypass using occipital artery graft as donor vessels [35]. We believe that presenting this distinct anatomy as 3D models as well as VR and AR can facilitate learning of such anatomy. Data in the literature shows that the utilization of 3D atlases helps students in the learning process, particularly in developing spatial memory, which is known to play an important role in surgery [19-21]. However, there is no solid evidence that the use of computer-based 3D atlases and VR atlases is more efficient than traditional teaching, despite the fact that a high percentage of students find the 3D atlases more helpful than traditional ones [7,8,19-21]. Stepan et al. [8] found that a VR experience was more engaging, enjoyable, and useful to students, compared with the 2D images that are commonly used in anatomical atlases. Chittaro et al. [4] stress that the VR type of anatomical study provides a more involving "first-person" experience in contrast to conventional 2D atlases, which the author refers to as a "third-person" experience requiring more cognitive effort. In another study, Kolla et al. demonstrate that VR learning is a less cognitively demanding method and is reported to be a better learning experience for medical students due to the "gamification" effect of the VR modality [19]. However, such studies are often performed at individual medical institutions, and 3D model-based learning has to be used in larger, comparative multi-institutional studies aiming to better evaluate the educational benefits of this technology [2]. Nevertheless, there are some negative aspects to using VR 3D for anatomical education, especially when some study participants complain of some adverse effects such as mild nausea, blurred vision and disorientation, tripping over wires connecting the VR device to the computer, walking into walls, etc. [8,19,25]. In our study, we found that the immersive VR experience is very useful for presenting the 3D models, but there were also issues with dizziness reported by some members of the team with prolonged use of the headset. Another problem that we found is that it was somehow difficult to read the annotations in the VR mode of the web-based platform. In the present study, we have not tested the benefits of 3D web-based models or AR/VR as a study tool for neurosurgery residents, but this is planned for future studies. The subjects who tested the technology (neurosurgical residents, board-certified and senior neurosurgeons, anatomists), including 3D web-based models, mobile phone/tablet AR and headset mount VR devices, all expressed their positive attitude and enthusiasm regarding the potential benefits that these modalities can add to neurosurgical and neuroanatomical education.

Other types of 3D models that are used for medical education and training are made by professional 3D artists, and the textures of the 3D models are not generated from photographs based on actual dissections but from complex computer algorithms [7,8]. We believe that photogrammetry will facilitate the generation of 3D models by non-professional 3D artists and will make 3D technology more available to the general medical community, which can potentially facilitate medical education.

## Conclusions

In the present study, we present our initial experience with the technique of photogrammetry in generating photorealistic 3D anatomical models. We find that such technology offers great possibilities for improving neurosurgical education in terms of presenting layered anatomical dissections and conveying difficult-to-understand ideas in a more comprehensive way. Nevertheless, further research is needed to explore the potential benefits and drawbacks of this method for neurosurgical and anatomical education.
